# The Emergence of Avian Orthoavulavirus 13 in Wild Migratory Waterfowl in China Revealed the Existence of Diversified Trailer Region Sequences and HN Gene Lengths within this Serotype

**DOI:** 10.3390/v11070646

**Published:** 2019-07-13

**Authors:** Yidong Fei, Xinxin Liu, Jiaqi Mu, Junjiao Li, Xibing Yu, Jin Chang, Yuhai Bi, Tobias Stoeger, Abdul Wajid, Denys Muzyka, Kirill Sharshov, Alexander Shestopalov, Alongkorn Amonsin, Jianjun Chen, Zhuang Ding, Renfu Yin

**Affiliations:** 1 Department of Veterinary Preventive Medicine, College of Veterinary Medicine, Jilin University, Xi’an Road 5333, Changchun 130062, China; 2 College of Food Science and Engineering, Jilin University, Xi’an Road 5333, Changchun 130062, China; 3 CAS Key Laboratory of Pathogenic Microbiology and Immunology, Institute of Microbiology, Chinese Academy of Sciences, Beijing 100101, China; 4 Comprehensive Pneumology Center, Institute of Lung Biology and Disease (iLBD), Helmholtz Zentrum Muenchen, Ingolstaedter Landstrasse 1, D-85764 Neuherberg/Munich, Germany; 5 Department of Biotechnology, Virtual University of Pakistan, 1-Davis Road, Lahore 54000, Pakistan; 6 National Scientific Center Institute of Experimental and Clinical Veterinary Medicine, 61023 Kharkiv, Ukraine; 7 Laboratory of modeling and monitoring of infectious diseases, Department of experimental models and pathogenesis of infectious diseases, Federal Research Center of Fundamental and Translational Medicine (CFTM), Novosibirsk 630117, Russia; 8 Center of Excellence for Emerging and Re-emerging Infectious Diseases in Animals, Faculty of Veterinary Science, Chulalongkorn University, Bangkok 10330, Thailand; 9 CAS Key Laboratory of Special Pathogens and Biosafety, Wuhan Institute of Virology, Chinese Academy of Sciences, Wuhan 430071, China; 10 National Virus Resource Center, Wuhan Institute of Virology, Chinese Academy of Sciences, Wuhan 430071, China

**Keywords:** *avian orthoavulavirus* 13, migratory waterfowl, HN gene, trailer, genetic relationships

## Abstract

*Avian orthoavulavirus 13* (AOAV-13), also named *avian paramyxovirus 13* (APMV-13), has been found sporadically in wild birds around the world ever since the discovery of AOAV-13 (AOAV-13/wild goose/Shimane/67/2000) in a wild goose from Japan in 2000. However, there are no reports of AOAV-13 in China. In the present study, a novel AOAV-13 virus (AOAV-13/wild goose/China/Hubei/V93-1/2015), isolated from a wild migratory waterfowl in a wetland of Hubei province of China, during active surveillance from 2013 to 2018, was biologically and genetically characterized. Phylogenetic analyses demonstrated a very close genetic relationship among all AOAV-13 strains, as revealed by very few genetic variations. Moreover, pathogenicity tests indicated that the V93-1 strain is a low virulent virus for chickens. However, the genome of the V93-1 virus was found to be 16,158 nucleotides (nt) in length, which is 12 nt or 162 nt longer than the other AOAV-13 strains that have been reported to date. The length difference of 12 nt in strain V93-1 is due to the existence of three repeats of the conserved sequence, “AAAAAT”, in the 5′-end trailer of the genome. Moreover, the HN gene of the V93-1 virus is 2070 nt in size, encoding 610 aa, which is the same size as the AOAV-13 strain from Japan, whereas that of two strains from Ukraine and Kazakhstan are 2080 nt in length, encoding 579 aa. We describe a novel AOAV-13 in migratory waterfowl in China, which suggests that diversified trailer region sequences and HN gene lengths exist within serotype AOAV-13, and highlight the need for its constant surveillance in poultry from live animal markets, and especially migratory birds.

## 1. Introduction

Viruses of the family *Paramyxoviridae* circulated in various vertebrates, including mammals, birds, reptiles and fish, have been newly identified [1,2]. Viruses of the subfamily *Avulavirinae*, previously designated avian avulaviruses (AAvV), are enveloped, non-segmented negative-sense single stranded RNA viruses, which are in the range of 14.9 to 17.4 kb in length, encode up to nine different proteins [3] and have the capacity to cause diseases with varying clinical manifestations in more than 200 wild and domestic bird species [4]. There are currently three genera (*Metaavulavirus*, *Orthoavulavirus* and *Paraavulavirus*) within *Avulavirinae*, including 20 described serotypes (-1 to -20), based on haemagglutination inhibition (HI) and neuraminidase inhibition (NI) assays and genetic sequencing [5,6,7,8,9,10]. In detail, *Metaavulavirus* contains ten species of *avian metaavulavirus* (AMAV), including serotypes -2, -5, -6, -7, -8, -10, -11, -14, -15 and -20, *Orthoavulavirus* contains eight species of *avian orthoavulavirus* (AOAV), including serotypes -1, -9, -12, -13, -16, -17, -18 and -19, while only two serotypes (-3 and -4) of *avian paraavulavirus* (APAV) are grouped into the genus of *Paraavulavirus* [11].

While little is known about the biological and genetic information of serotypes 2 to 20, extensive studies have been conducted on AOAV-1 (Newcastle disease virus, NDV) because of its high mortality and heavy losses on the economy in relation to the poultry industry worldwide [12,13]. AOAV-13 was first identified in wild goose feces in 2000 in Japan (AOAV-13/wild goose/Shimane/67/2000) [14] and has been sporadically isolated from migratory wild birds in Kazakhstan and Ukraine [15,16]. Migratory birds are known to be a natural reservoir of numerous avian viruses, including viruses within the subfamily, *Avulavirinae* [17]. Meanwhile, the potential for AOAV-13 dispersal and intercontinental transmission is high, because China is a very important region, where intercontinental flyways connecting the Russian Far East, Black Sea/Mediterranean, Alaska, Europe, Eastern Siberia, Eastern Mongolia and Asia cross each other, and hundreds of bird species, such as wild ducks, geese and swans concentrate and overwinter in wetlands during migration and breeding periods [18]. However, no study has reported on the molecular characteristics and genetic relationships of AOAV-13 in China, knowledge of which is essential for understanding the genetic diversity and epidemiology of the viruses of *Avulavirinae*.

## 2. Materials and Methods

### 2.1. Ethical States

All animal experimental protocols used in this study was approved by the Institutional Animal Care and Use Committee of Jilin University, China (approval number: 201803036, Date of approval: 8 March 2018).

### 2.2. Sample Collection and Virus Isolation

During the years from 2013 to 2018, both cloacal and oropharyngeal swab samples from clinically healthy domestic poultry from the live bird market (*n* = 3974) and fresh fecal samples from migratory birds in wetlands (*n* = 14,909) were collected from six provinces of China, including two provinces in Central China (Hubei and Hunan), one in East China (Anhui), one in Northeast China (Jilin), one in Northwest China (Qinghai) and one in North China (Neimenggu), as part of both the avian influenza virus (AIV) and NDV surveillance program [18,19,20,21]. The detailed information on sample collection, transportation, and handing as described in our previous study [18,19]. In brief, samples were collected using sterile swabs and placed into viral transport media containing 2 mg/mL streptomycin, 50 U/mL nystatin, 50 µg/mL gentamycin, 2000 U/mL penicillin, and 0.5% bovine serum albumin. Swabs were stored in liquid nitrogen during the fieldwork, and were kept at −80 °C after return to the laboratory.

Each individually collected swab specimen was inoculated into allantoic cavities of 9- to 10-day-old specific-pathogen-free (SPF) embryonated chicken eggs using standard techniques, as previously described for ND [22]. The present of the AOAV-13 in allantoic fluid was confirmed by seminested PCR and sanger sequencing for paramyxoviruses [23].

### 2.3. RNA Extraction, cNDA Synthesis, PCR and Whole Genome Sequencing

RNA extraction, cNDA synthesis, and seminested PCR and sanger sequencing for L gene of paramyxoviruses were performed according to the methods described in our previous study [20]. To explore the genetic characteristics of this V93-1 isolate, a complete viral genome sequence was determined by next-generation sequencing (NGS) [24], based on the random sequencing of total RNA using the de novo assembly method. Briefly, 2 mL of allantoic fluids containing V93-1 virus was used for RNA isolation using the TriPure Isolation Reagent (Roche, Shanghai, China), and then reverse transcription reactions were conducted using the protocols described in a previous study [20]. The sequencing library (using 1.5 ng purified cDNA) with an insert size of 200 bp was prepared by end-repairing, dA-tailing, adaptor ligation, and PCR amplification. The library was sequenced on an Illumina HiSeq 4000 platform, and genome assembly was done using the Galaxy platform interface [24,25].

### 2.4. Pathogenicity Test and Virus Infection of Cells

The pathogenicity test of the V93-1 virus, including the mean death time (MDT), the intra-cerebral pathogenicity index (ICPI), and intravenous pathogenicity index (IVPI), were determined according to the protocols described in the Office International des Epizooties (OIE) manual for ND [22]. Virus infection of MDCK, HD 11 and DF-1 cells with the presence or absence of TPCK-trypsin were performed according to the methods as previously described [20].

### 2.5. Virus Infection of Chickens

To further determine the biological characteristic of this V93-1 virus, eight 21-day-old SPF chickens were inoculated with a single dose of 100 µL allantoic fluid (HA titer of 1024 per 25 µL) via intraocular-nasal dropping. Then all chickens were monitored for clinical signals for 10 days. Cloacal and oropharyngeal swab samples were harvested at 2, 4, 7, and 10 dpi (days post infection) for the detection of virus shedding. In addition, at 2, 4, 7, and 10 dpi, two chickens were randomly sacrificed and tissue samples (including lung, kidney, liver, and spleen) and blood were harvested for the detection of virus load in vivo. For detection of virus shedding and virus load in infected chickens, seminested PCR for L gene of V93-1 virus was applied [23].

### 2.6. Phylogenetic Analysis

To understand genetic relationships of the V93-1 virus, phylogenetic trees were constructed based on the complete nt sequences of the genome and F gene of the AOAV-13 isolates, including V93-1 and other viruses of serotypes, using the maximum likelihood method based on the Tamura–Nei model [26]. The percentage of replicate trees in which the associated taxa clustered together in the bootstrap test (1000 replicates) are shown next to the branches. Initial tree(s) for the heuristic search were obtained automatically by applying Neighbor–Join and BioNJ algorithms to a matrix of pairwise distances estimated using the maximum composite likelihood (MCL) approach, and then selecting the topology with the superior log likelihood value. The tree is drawn to scale, with branch lengths in the same units as those of the evolutionary distances used to infer the phylogenetic tree. The evolutionary distances were computed using the maximum composite likelihood method and are in the units of the number of base substitutions per site. Codon positions included were 1st+2nd+3rd+Noncoding. All positions containing gaps and missing data were eliminated. Evolutionary analyses were conducted in MEGA X.

## 3. Results and Discussion

In this study, for the first time, we obtained a novel AOAV-13 virus (AOAV-13/wild goose/China/Hubei/V93-1/2015, V93-1 isolate, GenBank: MN150295) from migratory waterfowl at the Chenhu Lake Wetland of Hubei province (longitude 113.87, latitude 30.305), China, in 2015, and compared it to other available AOAV-13 isolates in GenBank to assess the biological characteristics and epidemiological genetic relationships of the V93-1 virus. In line with the reported studies [14,15,16], no domestic poultry origin AOAV-13 isolate was confirmed in this study. This may be because the AOAV-13 virus detection was neglected, as these viruses are negligible economically in relation to the poultry industry. Future AOAV-13 epidemiological surveillance studies should include more samples from poultry, must consider that their susceptibility to AOAV-13 viruses may differ from that of other avian species and that the infection could have an economic significance. Similar results were observed in a recent study, where the chicken-origin virulent AOAV-1 viruses infect chickens and efficiently transmit it to naïve birds, while the virulent viruses from wild birds, such as cormorant and pigeon, infect chickens only at higher doses and cannot transmit to other birds [27].

The V93-1 isolate was successfully propagated in 9- to 10-day-old SPF chicken embryos, and the harvested infective allantoic fluid had a hemagglutination (HA) titer of 512–1024 per 25 µL. Meanwhile, the V93-1 isolate had a high HI titer (1:512) for homologous V93-1 specific chicken antibodies, but low (less than 1:8) or no cross-reactivity with other serotypes, including AOAV-1, APAV-4 and AMAV-6. In addition, the mean death time (MDT) score in 9- to 10-day-old embryonated SPF chicken embryos was more than 168 h, with no mortality after 7 days, and both the intra-cerebral pathogenicity index (ICPI) in 1-day-old SPF chickens and the intravenous pathogenicity index (IVPI) in 6-week-old chickens were 0, suggesting that this V93-1 isolate is a low virulent virus for chickens.

To further determine the biological characteristic of this V93-1 virus, eight 21-day-old SPF chickens were inoculated with a single dose of 100 µL allantoic fluid (HA titer of 1024 per 25 µL) via intraocular-nasal dropping. However, no clinical signals were observed in virus-infected chickens during 2–10 days of post inoculation. Meanwhile, the viral load could not be detected in the organs (lung, kidney, and spleen) and blood of the experimentally infected chickens, and cloacal, oropharyngeal swab samples from the infected chickens were found to be negative by PCR. In addition, the virus did not cause a cytopathic effect without exogenous protease in tested MDCK, HD 11 and DF-1 cell lines. However, the growth and other biological characteristics need to be detected with other cell lines.

**Figure 1:**
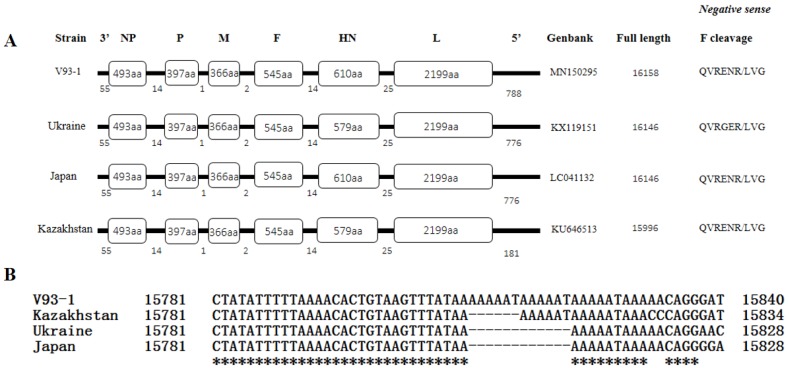
Genetic map (**A**) and alignment of the trailer regions (**B**) of AOAV-13 isolates used in the study. (**A**) Individual gene ORFs are indicated by rectangles. The amino acid length of each encoded protein is shown in each box, and the nucleotide (nt) lengths of the non-coding leader, intergenic, and trailer regions are shown under each map, and GenBank accession number, the total genome nt length and the sequence for the F0 protein cleavage site are shown in parentheses to the right. (**B**) Asterisk indicate identities and short-term indicate difference.

The genome of the V93-1 virus was found to be 16,158 nucleotides (nt) in length, with a GC content of 42.6%, which is 12 nt longer than those of strains from Japan (GenBank: LC041132) and Ukraine (KX119151) and 162 nt longer than the strain from Kazakhstan (KU646513) (Figure 1A). The genome of V93-1 encodes seven putative proteins (NP, P/V, M, F, HN, and L) in the following order: 3′ leader-NP-P/V-M-F-HN-L-trailer 5′ (Figure 1A). Upon comparison with the sequences available from the GenBank database, the V93-1 virus has a 97–98% whole genome nucleotide sequence similarity to the AOAV-13 strains isolated from Ukraine, Japan and Kazakhstan, while only 72% to AOAV-1 (KR732614, 462/639). Interestingly, the HN gene of the V93-1 virus is 2070 nt in size (ORF, 1833 nt), encoding an HN protein of 610 aa, which is the same in size as the strain from Japan. In contrast, the HN gene of strains from Ukraine and Kazakhstan is 2080 nt in length (ORF, 1740 nt), encoding an HN protein of 579 aa (Figure 1A). The entire sequence of the HN gene of the V93-1 virus was confirmed by sanger sequencing. Meanwhile, the HN protein of the strain, V93-1, has a high level of aa sequence identity with the HN proteins of the strains from Japan (98%, 600/610), Kazakhstan (99%, 575/579) and Ukraine (99%, 573/579), and a lower level of aa sequence identity (≤60%) to other serotypes, such as AMAV-2 and AOAV-1, when compared with the aa sequences available from the GenBank database. Furthermore, the length of the 5′ trailer region of the V93-1 virus was 787 nt, which is 607 nt longer than the strain from Kazakhstan and 12 nt longer than those of strains from Japan and Ukraine (Figure 1A). In addition, a conserved sequence, “AAAAAT”, was found in the 5′ trailer region of AOAV-13 [14,15,16], but not in the other serotypes. By comparison, V93-1, obtained in the year 2015, has three repeats of “AAAAAT” (Figure 1B), while the isolate from Kazakhstan, obtained in 2013, has two repeats, and isolates from Japan (2010) and Ukraine (2011) have only one repeat. However, all reported AOAV-13 isolates are avirulent, suggesting that the number of the repeats of “AAAAAT” is irrelevant to the virulence of AOAV-13. Nonetheless, additional studies on the roles of the conserved sequence, “AAAAAT,” to AOAV-13 propagation should be included in the future. The presence of three repeats of “AAAAAT” within the 5′ trailer region of the V93-1 virus was also confirmed by sanger sequencing.

**Figure 2:**
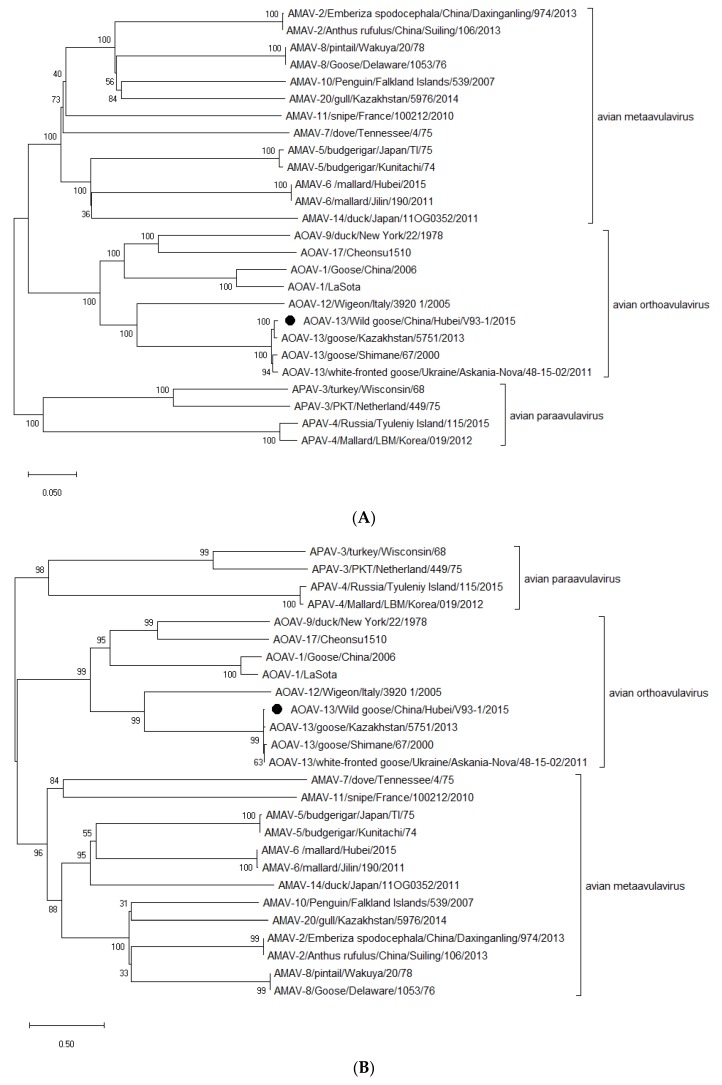
Phylogenetic analysis of the whole genome sequence (**A**) and entire F gene sequences (**B**) of AOAV-13 and other serotype viruses. In line with other studies [5,6,7,8,9,10], three genera (*Metaavulavirus*, *Orthoavulavirus* and *Paraavulavirus*) exist in the subfamily *Avulavirinae*, including 20 described serotypes (-1 to -20), as revealed by genetic relationships of the whole genome sequence (**A**) and entire F gene sequences (**B**) of all viruses within *Avulavirinae*. As shown in Figure 2A,B, *Metaavulavirus* contains ten species of avian metaavulavirus (AMAV), including serotypes -2, -5, -6, -7, -8, -10, -11, -14, -15 and -20, *Orthoavulavirus* contains eight species of avian orthoavulavirus (AOAV), including serotypes -1, -9, -12, -13, -16, -17, -18 and -19, while only two serotypes (-3 and -4) of avian paraavulavirus (APAV) are grouped into the genus of *Paraavulavirus* [11]. The evolutionary history was inferred by using the maximum likelihood method based on the Tamura–Nei model. The optimal tree with the sum of branch length of 3.61319067 (**A**) and 21.37661970 (**B**) is shown. The analysis involved 26 (**A**) and 26 (**B**) nucleotide sequences. There were a total of 12,066 (**A**) and 1414 (**B**) positions in the final dataset.

To further determine the genetic relationships of the V93-1 virus, phylogenetic trees were constructed, based on the complete nt sequences of the genome and F gene of the AOAV-13 isolates, including V93-1 and other viruses of serotypes (Figure 2). Phylogenetic analysis of the whole genome and F gene of V93-1 revealed a very close genetic relationship among these AOAV-13 strains and showed very few genetic variations. However, V93-1 showed a closer relationship to the strain recently isolated from Kazakhstan, when compared to the strains isolated from Japan and Ukraine. This may be explained in terms of the migration of wild birds into Hubei province of China from Kazakhstan, as three large flyways of migratory waterfowl, including Black Sea/Mediterranean, East Asian/Australian and Central Asian waterfowl, where cases of reassortment and intercontinental spread among many avian viruses, such as AOAV-1 and avian influenza viruses, in China and other geographic regions, have been detected [17,18,19].

## 4. Conclusions

To our knowledge, our study is the first to describe an AOAV-13 isolate in wild migratory waterfowl from China, demonstrating that not only does the V93-1 virus harbor the longest genome size (16,158 nt) and three repeats of the uniquely conserved sequence (AAAAAT) within the 5′ trailer, as compared to the three previously reported completely sequenced isolates, but also at least two different HN proteins, which exist within the serotype AOAV-13. Furthermore, the emergence of the V93-1 virus in China is epidemiologically connected to the AOAV-13 from other geographical regions, such as Kazakhstan. Therefore, its presence signified a potential risk of AOAV-13 being introduced into the territory and country. It also necessitates the demand for constant epidemiological surveillance, including geographic, ecological and/or host-species factors for AOAV-13 isolates among wild birds and domestic poultry in China, to prevent and control the potential transmission of novel variants from other regions.
